# Different strategies of bipeds and quadrupeds to maintain postural stability- a comparison of healthy humans and dogs via static posturography

**DOI:** 10.1038/s41598-026-42726-2

**Published:** 2026-03-09

**Authors:** Masoud Aghapour, Nadja Affenzeller, Christiane Lutonsky, Christian Peham, Barbara Bockstahler

**Affiliations:** 1 Physical Therapy, Clinical Centre for Small Animal Health and Research, Clinical Department for Small Animals and Horses, University of Veterinary Medicine (vetmeduni), Vienna, Austria; 2 Behavioural Medicine, Clinical Centre for Small Animal Health and Research, Clinical Department for Small Animals and Horses, University of Veterinary Medicine (vetmeduni), Vienna, Austria; 3 Movement Science Group, Clinical Centre for Equine Health and Research, Clinical Department for Small Animals and Horses, University of Veterinary Medicine (vetmeduni), Vienna, Austria

**Keywords:** Center of pressure, Gait analysis, Posturography, Ground reaction forces, Romberg Index, Animal physiology, Biomechanics

## Abstract

Postural stability (PS) is the ability to maintain balance through the integration of sensory inputs from the proprioceptive, visual, and vestibular systems. This study compared PS between healthy adult humans and dogs using static posturography and investigated the effect of blindfolding on PS. Twenty-two healthy young adults and twenty-two healthy young pet dogs were assessed using a pressure measurement platform under eyes open and eyes closed conditions. The Romberg indices of 5 center of pressure (COP) parameters were calculated: mediolateral displacement (RI MLD%), craniocaudal/anteroposterior displacement (RI CCD%), COP length (RI L%), average speed (RI AS), and support surface (RI SS%). Significant differences were observed between humans and dogs for RI CCD%, RI L%, and RI AS. Humans exhibited greater reliance on visual input for balance, indicated by higher RI values for these parameters, suggesting increased instability when blindfolded. In contrast, dogs showed lower RI values, indicating less reliance on vision and more stable postural control. The lack of significant differences in RI MLD% and RI SS% suggests similar mediolateral and support surface compensatory mechanisms between species. These findings reveal fundamental mechanistic differences in how balance is maintained between the two species.

**Supplementary Information:**

The online version contains supplementary material available at 10.1038/s41598-026-42726-2.

## Introduction

Postural stability (PS) refers to the ability to achieve, maintain, or restore balance during various postures and activities^1^. PS relies on the precise integration of sensory inputs from the proprioceptive, visual, and vestibular systems, which are processed and coordinated by the central nervous system^2,3^. This intricate interplay allows for muscle contractions that counteract gravity, enabling controlled movements and maintaining the body’s center of mass (COM) within its base of support (BOS)^1,4–8^. The BOS refers to the surface area defined by the contact points between the body and the ground^9^.

Posturography is a method employed to evaluate postural control under static^10–16^ and dynamic^15,17–22^ conditions which is mostly based on the evaluation of the spatiotemporal statistics of the center of pressure (COP) of the whole body or each foot/limb. In quiet stance, the COP moves around the projection of the COM. The COP is the point location of the ground reaction force (GRF), which is used by the body to control balance around the vertical projection of the COM^7,23^. Analyzing various COP parameters provides valuable insights and has been a focus of research in both human^23,24^ and veterinary medicine^10,13,25^.

Static posturography is widely utilized in clinical practice to diagnose balance disorders, track rehabilitation progress, and evaluate the efficacy of treatments for balance-related conditions. In addition, it is frequently used in research projects to investigate the performance of postural control in various populations. Lower COP values such as anterorposterior (craniocaudal in dogs) and mediolateral displacement of the COP as well as average speed of the COP are indicative of better stability in static posturography, as they reflect reduced COP displacement, suggesting better controlled and more stable postural control mechanisms^10,23,26^. This makes static posturography an essential tool in both clinical and research settings for understanding and improving balance across species. For instance, in addition to research in humans^16,27^, this method has been applied across various species, ranging from dogs^10,15,25,26,28^ and ponies^13^ to horses^14,29^ and even wild animals such as elephants^30^ and rats^31^. Conventional COP parameters explored in the literature include anterorposterior (craniocaudal in dogs) and mediolateral displacement of the COP, total length of COP excursion, average speed, and the support surface of the COP^16,19–21,23,32^.

As an active process, postural balance supports essential activities such as standing and walking by continuously adjusting body position and orientation in response to sensory feedback. Impairments in these systems can disrupt PS, underscoring the importance of studying this complex mechanism to better understand how sensory and motor systems interact to maintain physical stability and adaptability. However, it should be noted that standing (static balance) differs from dynamic tasks such as walking (dynamic steady-state balance) or responses to perturbations (dynamic reactive balance), and findings obtained under static conditions may not directly translate to dynamic situations^33^. The effect of various external perturbations, including mechanical and sensory disturbances, has been investigated in veterinary^29,34^ and human medicine^35–37^.

Despite numerous studies in both veterinary and human medicine, cross-species comparisons, particularly between humans and dogs, are limited^5^. Cross-species comparisons, such as between humans and dogs, are valuable for understanding fundamental principles of balance and postural control, as many neural and musculoskeletal mechanisms are conserved across mammals^30,31,38,39^. Differences in body structure and posture, such as bipedal versus quadrupedal stance, also highlight how species-specific adaptations influence stability^30,39^. Insights from such comparisons can inform rehabilitation strategies, guide the design of assistive devices, and inspire bioinspired approaches in robotics^40^. Such comparisons can be challenging due to differences in equipment and methodologies used in human and animal research, as well as anatomical and anthropometric disparities that complicate this comparison (e.g., bipedal vs. quadrupedal stance). Conducting experiments in animals is already difficult, and this challenge becomes even greater when working with wild animals e.g., elephants^30^, flamingos^41^, and rats^31^, where training these animals to stand completely still during static posturography experiments can be both difficult and, in some individuals almost impossible. Thus, efforts have been made to conduct these examinations in the shortest time possible. However, differences in experimental setups and protocols often result in inconsistent outcomes. Therefore, using standardized protocols is essential for obtaining comparable data. A recent validation study of static posturography in dogs reported that one 10-second measurement or two 5-second measurements yielded the shortest duration with highly reproducible results for all 5 of the investigated COP parameters^32^.

The reason for choosing dogs in this study is that they are one of humans’ oldest companion animals, making them more readily accessible and easier to work with in research settings compared to other species. Static posturography requires subjects to stand completely still for several seconds, which is challenging in many animals; for instance, motion analysis in cats is often more difficult than in dogs because keeping them stationary, even for a short time, can be unpredictable and requires training to teach them to stand on the force platform^42^. Similar challenges are amplified when working with wild animals^30,31,41^. Dogs, however, can be trained more reliably to remain still, allowing for accurate and reproducible measurements within short time frames^43^. Studying postural control in dogs not only facilitates cross-species comparisons with humans but also has direct applications in canine physical medicine^44,45^. Future studies could extend these methods to other domestic or large animals, such as horses, to broaden comparative insights into postural control across species.

A valuable tool for the assessment of PS and balance, particularly in neurological examinations, is the Romberg index (RI)^27,46,47^. This index measures the degree of sway or movement when an individual stands with their eyes open versus closed, providing insights into the sensory contributions to balance. Therefore, the RI is a valuable tool to compare postural stability also between species because it offers a standardized method to evaluate the reliance on visual cues for maintaining balance. By comparing the RI across species, we aim to understand the differences in sensory integration and balance mechanisms. This comparison is crucial for identifying unique adaptations in canine balance and for developing cross-species insights into balance-related disorders.

In this study, we aimed to compare the PS of adult healthy humans and adult healthy pet dogs by using static posturography and investigate the effect of the absence of the visual input (when blindfolded) on PS between species. We hypothesized that the RI differs between humans and dogs. We estimated the corresponding RI values to quantify these differences in relation to anthropometric characteristics and species-specific reliance on visual input.

## Results

The results of the descriptive statistics of the RI for humans and dogs are presented in Table 1. The RI values calculated from normalized mediolateral displacement of the COP (RI MLD%) in humans were below 100 (RI MLD% = 95.26 ± 39.66), indicating reduced reliance on visual input for maintaining balance and suggesting increased postural stability when vision was removed. In contrast, dogs showed RI MLD% above 100 (RI MLD% = 113.27 ± 32.93), reflecting greater reliance on visual information to maintain balance and increased postural instability when vision was removed.

**Table 1:** Descriptive statistics of the Romberg indices for humans and dogs.

Parameter^*^	Groups	Mean	Standard Deviation	95% Confidence Interval of the Mean	
				Lower bound	Upper bound
RI MLD%	Human	95.26	39.66	77.68	112.84
	Dog	113.27	32.93	98.67	127.87
RI CCD%	Human	113.83	43.84	94.39	133.26
	Dog	87.74	24.93	76.69	98.79
RI L%	Human	110.71	25.11	99.58	121.84
	Dog	92.26	25.55	80.93	103.58
RI AS	Human	114.98	20.20	106.02	123.94
	Dog	88.68	24.63	77.76	99.61
RI SS%	Human	111.23	70.70	79.88	142.58
	Dog	108.48	52.10	85.38	131.58

*RI MLD%: Romberg Index calculated from normalized mediolateral displacement of COP; RI CCD%: Romberg Index calculated from the normalized anterorposterior (craniocaudal) displacement of COP; RI L%: Romberg Index calculated from the normalized length of COP; RI AS: Romberg Index calculated from the average speed of COP movement; RI SS%: Romberg Index calculated from the normalized support surface of COP.

Contrary to the RI MLD%, the results for all other RI values were higher in humans compared to dogs, indicating greater postural instability in humans when vision was removed. Specifically, the RI CCD%, RI L%, and RI AS values in humans were above 100, whereas the corresponding values in dogs were all below 100. For RI SS%, the values reported for humans were also higher than those for dogs; however, both groups showed values above 100, indicating increased postural instability when blindfolded.

**Fig. 1:**
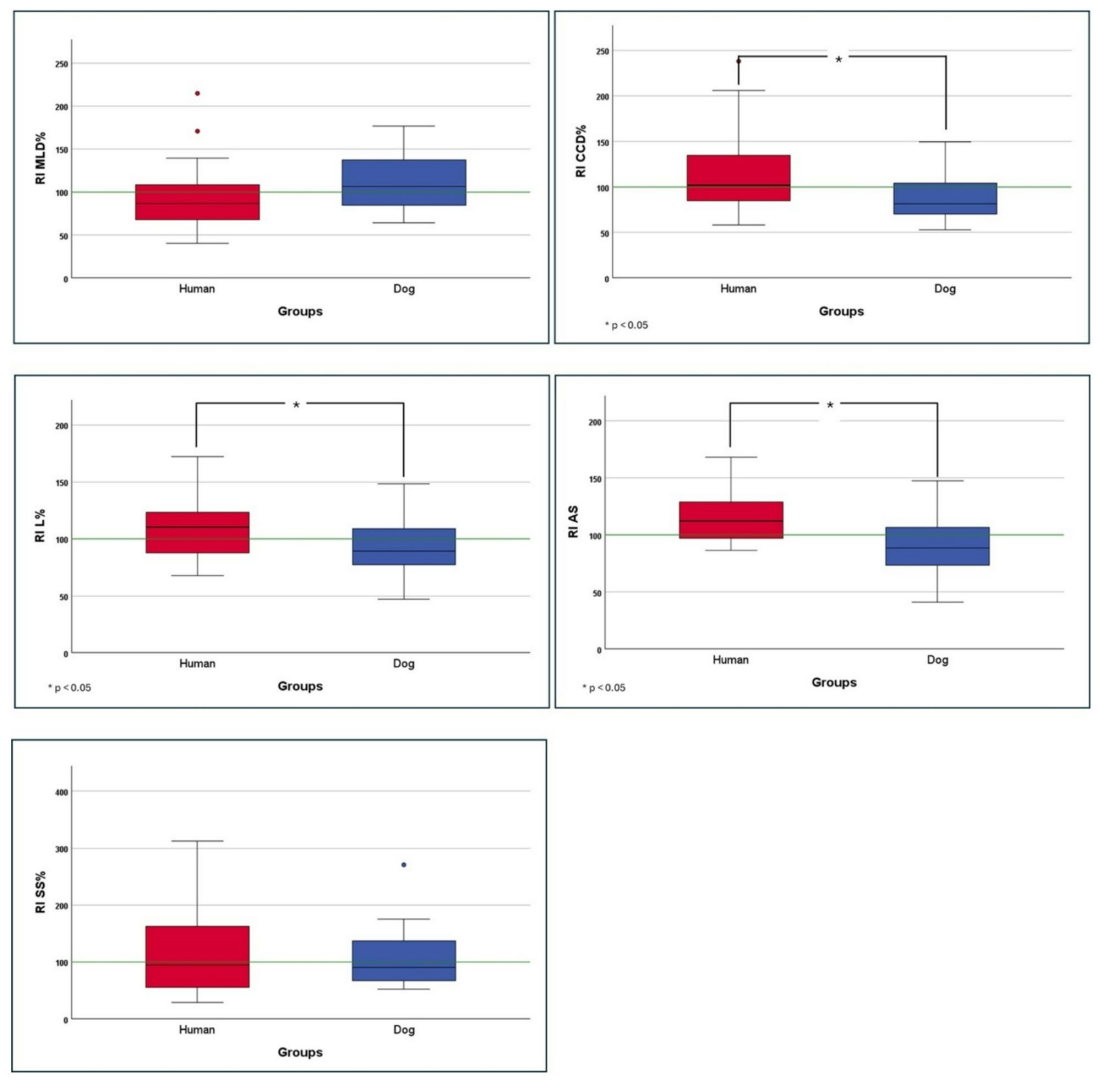
Comparison of Romberg Index (RI) parameters between humans and dogs. RI MLD%: RI calculated from normalized mediolateral displacement of COP; RI CCD%: RI calculated from normalized anteroposterior (craniocaudal) displacement of COP; RI L%: RI calculated from normalized length of COP; RI AS: RI calculated from average speed of COP movement; RI SS%: RI calculated from normalized support surface of COP. The green line represents RI = 100; equal performance with eyes open and eyes closed corresponds to this value, while deviations indicate the relative contribution of visual input to maintaining postural stability. Asterisks (*) indicate significant differences between groups (*p < 0.05).

Table 2 shows the outcomes of the Independent Samples t-test. All Levene’s test p-values were > 0.05, indicating that the assumption of equal variances was met. Significant differences (*p* > 0.05) between humans and dogs were found for RI CCD%, RI L%, and RI AS with humans showing higher values, based on one-way ANOVA (Table 3). No significant differences were found for RI MLD% and RI SS% between humans and dogs in this study (*p* = 0.11 and *p* = 0.88, respectively). Corresponding results for the RI parameters are presented in Fig. 1. However, further information regarding the COP and BOS parameters is provided in the Supplementary Materials.

**Table 2:** Independent samples t-test calculations of the romberg indices for humans and dogs.

Parameter^*^	Levene’s Test for Equality of Variances	
	F	Sig.
RI MLD%	0.01	0.93
RI CCD%	3.42	0.07
RI L%	0.00	0.98
RI AS	0.86	0.36
RI SS%	1.88	0.18

*RI MLD%: Romberg Index calculated from normalized mediolateral displacement of COP; RI CCD%: Romberg Index calculated from the normalized anterorposterior (craniocaudal) displacement of COP; RI L%: Romberg Index calculated from the normalized length of COP; RI AS: Romberg Index calculated from the average speed of COP movement; RI SS%: Romberg Index calculated from the normalized support surface of COP.

Statistical significance was considered at an alpha level of *p* < 0.05.

**Table 3:** One-way ANOVA results for of the romberg indices for humans and dogs.

Parameter^*^	F	*p*-value	Effect size	
			Omega-squared (ω²)	Interpretation
RI MLD%	2.69	0.11	0.04	Small effect
RI CCD%	5.89	0.02 ^†^	0.10	Medium effect
RI L%	5.84	0.02 ^†^	0.10	Medium effect
RI AS	14.99	< 0.001 ^†^	0.24	Large effect
RI SS%	0.02	0.88	−0.02	no effect

*RI MLD%: Romberg Index calculated from normalized mediolateral displacement of COP; RI CCD%: Romberg Index calculated from the normalized anterorposterior (craniocaudal) displacement of COP; RI L%: Romberg Index calculated from the normalized length of COP; RI AS: Romberg Index calculated from the average speed of COP movement; RI SS%: Romberg Index calculated from the normalized support surface of COP; ω² (Omega-squared) indicates the proportion of variance in the dependent variable explained by the factor, corrected for sample error. Its values range from 0, indicating no effect, to 1, indicating that all variance is explained by the factor. According to Cohen (1988), ω² values of 0.01 to 0.06 are conventionally interpreted as a small effect, values from 0.06 to 0.14 as a medium effect, and values of 0.14 or higher as a large effect.

†The mean difference is significant at the 0.05 level.

## Discussion

This study aimed to evaluate the impact of loss of vision by blindfolding on PS within and between healthy adults and dogs while standing on a pressure platform. We hypothesized that the RI differs between humans and dogs. This hypothesis was supported by the results.

Our results revealed significant differences in the RI of three COP parameters (RI CCD%, RI L%, and RI AS) between humans and dogs, while no differences were observed for RI MLD% and RI SS%.

As previously mentioned, the RI quantifies the influence of visual input on PS^27,46,47^. A higher RI (RI > 100) indicates a greater reliance on visual information to maintain balance, suggesting increased postural instability when visual inputs are removed^16^. Conversely, a lower RI (RI < 100) indicates reduced dependence on visual input for PS. The threshold of 100 is conventionally used in the RI because it represents a balance point between visual and non-visual contributions. This threshold is derived from the original Romberg test paradigm, where equal performance with eyes open and eyes closed corresponds to an RI of 100, and deviations from this value reflect the relative contribution of visual input to maintaining PS^27^. Our results revealed species-specific differences in the RIs calculated for humans and dogs. Interestingly, for the RI CCD%, RI L%, and RI AS—parameters where significant interspecies differences were observed—a clear pattern emerged. For humans, the RIs (mean values) exceeded 100, indicating a greater reliance on visual information for balance, and suggesting increased postural instability when blindfolded. In contrast, the same RIs (mean values) for dogs were below 100, indicating a lower reliance on visual input for balance when blindfolded compared to humans.

**Fig. 2:**
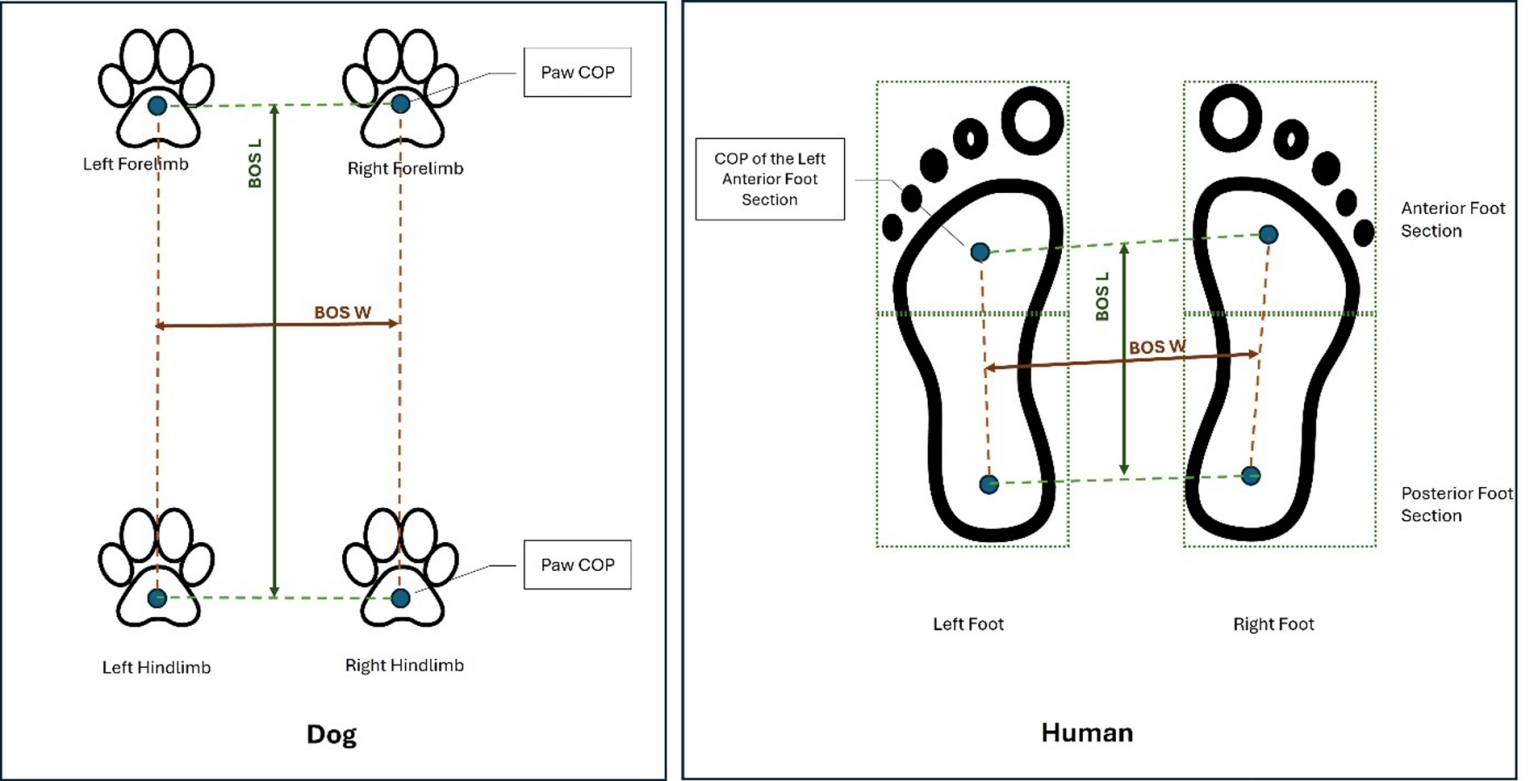
Schematic illustration of the method used to determine the length and width of the base of support in humans and dogs.

Despite RI CCD%, RI L%, and RI AS showing values above 100 in humans and below 100 in dogs, the RI MLD% values were below 100 in humans and above 100 in dogs, with no significant difference between the two species for this parameter under the tested conditions. Visual deprivation under these conditions may be associated with similar compensatory strategies in the mediolateral direction; however, this cannot be confirmed without a non-inferiority analysis. Furthermore, the relatively large standard deviations observed for RI MLD% in both humans and dogs indicate substantial interindividual variability, which may have reduced the ability to detect small inter-species effects. In a study on older adults, it was indicated that older adults depend more heavily on visual input to adjust mediolateral sway^48^. Another investigation involving adults examined COP parameters under both eyes open (EO) and eyes closed (EC) conditions. The findings indicated that various COP indicators are influenced by distinct neuromuscular and biomechanical processes that contribute to postural control^49^. Notably, the study revealed that maintaining anteroposterior stability requires the neuromuscular system to increase its effort by approximately 50% when visual input is removed^49^. Another important factor that may explain the observed interspecies differences is the shape of the BOS (Fig. 2). In dogs, the BOS is typically rectangular, with its length greater than its width. This configuration provides more stability in the craniocaudal (anteroposterior) direction but less in the mediolateral direction. Consequently, when visual input is removed, dogs may be more vulnerable to instability laterally, as suggested by their RI MLD% values exceeding 100, indicating increased postural sway. This interpretation is in line with previous reports describing increased mediolateral instability in dogs under challenging conditions^34^. In contrast, humans standing bipedally have a BOS that is relatively square or slightly rectangular. These BOS-related differences need to be investigated in future studies to better understand how fundamental biomechanical configurations, in addition to sensory reliance, shape species-specific balance control strategies.

**Fig. 3:**
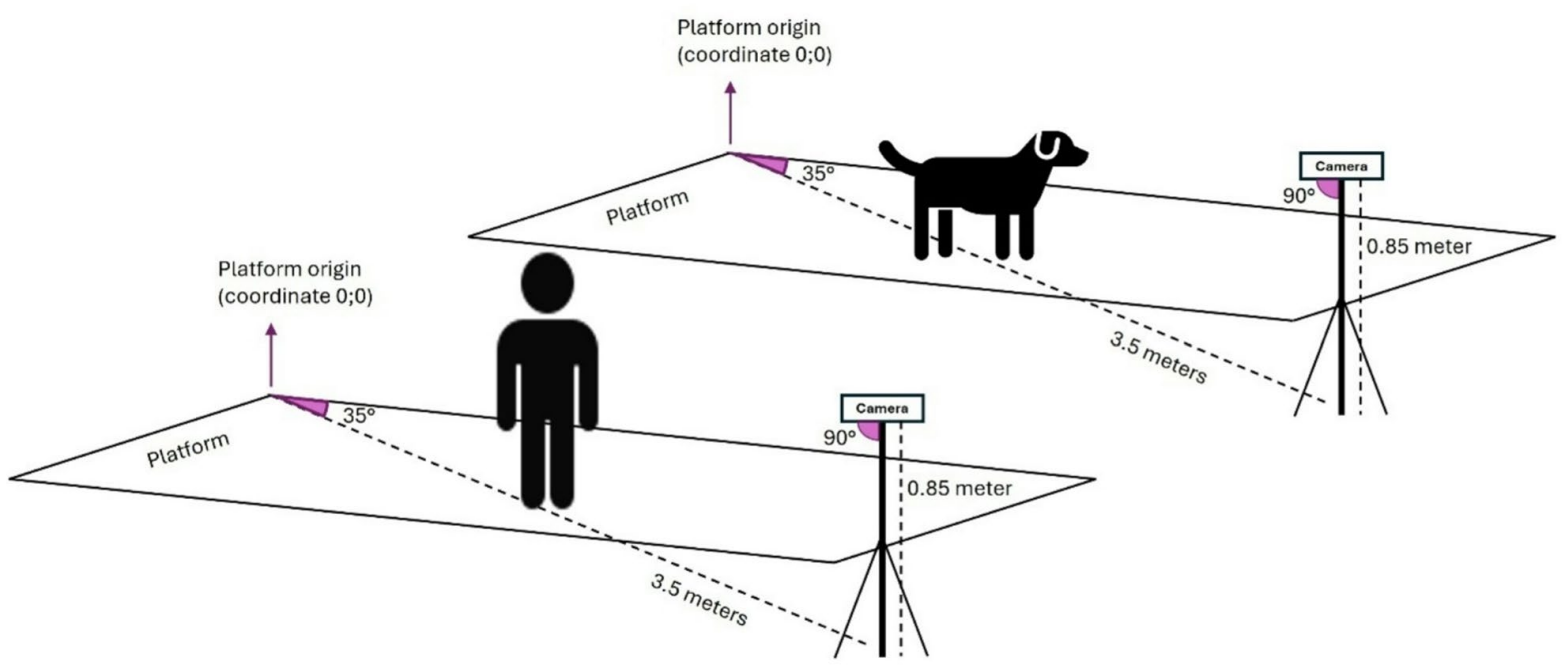
Schematic representation of the camera setup employed in the study with adult participants and dogs.

Similarly, the RI SS% did not differ significantly between humans and dogs, indicating that the loss of vision affects overall stability equally in both species. This might be because the support surface area is a more generalized measure of stability and is less influenced by species-specific control strategies or anatomical differences between bipeds and quadrupeds. In contrast, the other RI parameters capture more dynamic aspects of balance control. Significant differences in RI CCD%, RI L%, and RI AS suggest that humans and dogs employ different strategies for craniocaudal (anterorposterior) stability, the length of COP movement, and the speed of COP movement to compensate when visual input is removed. These differences could be attributed to the distinct anatomical structures and evolutionary adaptations of each species. Humans, being bipedal, may have more pronounced anterorposterior sway and different movement patterns compared to quadrupedal dogs, who have a lower COM and a wider BOS^5,50^.

When comparing the COP parameters of humans and dogs obtained through static posturography, it is crucial to consider the anatomical, physiological, and biomechanical differences between humans (bipeds) and dogs (quadrupeds). One of the most significant factors is the varying height or location of the COM or center of gravity (COG). The COM can be described as the theoretical point within the body where its mass is evenly distributed in all directions^50,51^. The terms COM and COG are often used interchangeably on Earth because, under the influence of Earth’s relatively uniform gravitational field, they coincide.

In humans, during quiet bipedal standing, the COM is typically located in the trunk, approximately one meter above the ankles^5^. In contrast, the COM in dogs during standing (with all extremities perpendicular to the surface) is lower than in humans. The COM in dogs is approximately located within the thoracal region. Specifically, it is near the xiphoid process (the lower part of the sternum), on the left side at 59% of the width of the chest, and 41% of the distance from the most dorsal to the most ventral aspect of the body^50^. This places the COM around the area of the thorax, approximately between the 8th and 10th ribs in a lateral view. It has also been noted that the 3D location of the COM in dogs can be influenced by the subject’s morphometrics^50^.

Given that, during quiet standing, the vertical projection of the COM represents the instantaneous vector of vertical GRF, a higher COM position may lead to greater COP sway, potentially reducing balance stability—aligning with biomechanical principles^30^. However, this conclusion has been challenged by another study^5^. It has been reported that despite differences in anthropometric parameters and the location of the COM in humans and different animals such as horses^29^, and rats^52^, COP oscillations were similar^5^. From our perspective, such conclusions may be an oversimplification, and more precise studies are needed for accurate comparisons.

When comparing studies on static posturography, it is crucial to consider various aspects to ensure the validity and comparability of the results. Firstly, the equipment used in the studies should be technically comparable. For instance, results acquired using a pressure platform cannot be directly compared with those obtained from a force plate^28^. Another important consideration is the study design. Since there is no standard guideline for static posturography, different studies may employ various procedural setups and methodologic designs, which can be even more diverse when comparing different species of animals (e.g. flamingos^41^ vs. elephants^30^. The duration of the measurement, the number of valid trials required, and the analysis of the acquired results are all critical factors that influence the outcomes^32^. When comparing such studies, it is essential to ensure that the same protocols were used in both studies. For example, it is important to verify whether the data were put through a filtering process before analysis and whether the mediolateral and craniocaudal sway of the COP was normalized to the BOS, as different animals have varying BOS width and length. For instance, in a study on dogs, it has been demonstrated that the morphology of dogs affects COP values, and PS is influenced by morphometric parameters^26^.This normalization is particularly important when comparing bipedals and quadrupedals. This is the central reason we are confident that our comparison of the two species is not only valid and methodologically robust, but also offers uniquely valuable insights. Additionally, the duration of the measurement can significantly impact the results. Comparing a longer measurement duration (e.g., 20 s) with a shorter one (e.g., 1 s) may not be appropriate, as confirmed by previous studies^32^.

Another point to consider is the analysis of the acquired data and inter-observer differences. Selecting an appropriate section for analyzing the acquired COP data may depend on the observer, making clear definitions and standardized protocols essential for this purpose. In an inter-observer study, a high correlation coefficient between observers has been reported for the analysis of COP data obtained from static posturography in healthy adult dogs^32^. However, it is important to note that in this study, all observers were trained in the protocols and had similar experience in data analysis. Future studies should focus on evaluating inter-observer reliability across different observers with varying levels of experience, from different teams. An important consideration here is the analysis of data acquired from different animal species, as certain definitions may vary depending on the type of animal. For instance, the observer-based definition of “standing still” may differ for wild animals compared to domestic animals or humans. Thus, precise definitions and detailed protocols for each animal type should be established to ensure accurate and consistent analysis.

Beyond the design and setup of studies, differences in the anatomy and physiology of animals and humans play a crucial role. For instance, when comparing COP data from rats^52^ and elephants^30^, anatomical differences must be taken into account. Previous studies suggest that sensorimotor control in large animals, such as elephants, is more complicated than that in smaller animals^30,53^. It has been reported that larger animals require more time to respond to external stimuli^53^. As body size increases, so does sensorimotor delay, which arises from multiple factors, including sensory perception, neural transmission, synaptic processing, muscle activation, and force production. As a result, both inertial effects and delayed neuromuscular responses contribute to greater difficulty in maintaining balance, particularly when the COM is positioned higher^30^.

Considering the factors mentioned above, we aimed to maintain consistency in protocols and setups for both humans and dogs in this study. The same measurement platform, software, and observers were used, following a standardized analysis protocol. In our previous study with the same design and protocols, a high inter-observer reliability was reported^32^. Additionally, all observers had a comparable level of expertise in acquiring and analyzing kinetic data. Furthermore, given the differences in BOS, anthropometric characteristics, and biomechanical differences between humans and dogs (bipedal vs. quadrupedal stance), mediolateral and craniocaudal (anterorposterior) COP sway was normalized to BOS width and length, respectively. Additionally, both the COP path length and the support surface of the COP were adjusted relative to the BOS.

When comparing static posturography between humans and dogs, it is essential to consider several factors, including the relative importance of sensory inputs, evolutionary adaptations, and the effects of blindfolding. Both species rely on visual, vestibular, and proprioceptive inputs for maintaining balance^1^. However, the relative importance of these inputs may differ between humans and dogs. On the other hand, evolutionary adaptations have significantly shaped the balance and postural control systems in both species. Humans have evolved to walk upright, which has influenced their balance strategies, such as the use of ankle, hip, and stepping strategies^54^. In contrast, dogs have evolved to be agile and fast runners, relying on different limb coordination and body adjustments to maintain stability. Blindfolding affects both species by removing visual input, a crucial component of their balance systems^48,49^. Based on the result of this study blindfolding leads to increased reliance on vestibular and proprioceptive inputs, resulting in increased sway and instability in humans. Dogs, with their lower COM/COG and larger BOS, seem to be less dependent on visual input with better adapted compensatory mechanisms, such as enhanced limb coordination and muscle activation when blindfolded, to maintain balance. In summary, anatomical differences, sensory input reliance, postural control strategies, compensatory mechanisms, and evolutionary adaptations are key points to consider when comparing static posturography between humans and dogs. Blindfolding highlights the importance of visual input and the different adaptive responses of both species to maintain stability.

In the current study, we used a pressure platform that measures only vertical GRF and does not capture shear forces, which may be considered a limitation. Differences in shear forces between conditions could potentially influence PS and balance strategies, particularly in dynamic situations. However, our study focused on static posturography, where vertical forces are the primary component of interest and have been widely accepted as reliable indicators of PS in both humans and animals. While shear forces may provide additional insights, especially in translational or dynamic balance tasks, their absence does not invalidate our findings but might restrict their interpretation to the vertical component of GRF.

Another limitation of this study is the use of 5-second trial segments for human participants. This duration was selected to maintain consistency with prior studies conducted in dogs^32^, facilitating cross-species comparisons. Although validation studies indicate that 5-second trials are sufficient for dogs^32^, it is not yet established whether this duration is optimal or fully valid for assessing behaviors or responses in human participants. Future research should investigate whether different trial durations might yield more reliable or sensitive measures in humans. Consequently, the present findings should be interpreted with this methodological consideration in mind.

When performing measurements in the EC condition, our human participants were asked to simulate looking at the same spot with their eyes closed, whereas in dogs, blindfolding was achieved using taped laser goggles. This difference might raise methodological concerns, as taped goggles may still allow partial vision from below the frame. Even though taped laser goggles used in dogs were carefully checked prior to use to ensure proper functioning (no vision), the most rigorous approach would be to standardize the method across species, for example, by having human participants wear similar goggles. This should be considered in future studies.

In conclusion, this study aimed to evaluate the impact of blindfolding on PS in humans and dogs using static posturography. The results revealed significant differences in the Romberg indices for RI CCD%, RI L%, and RI AS between humans and dogs, while no significant differences were observed for RI MLD% and RI SS%. These findings suggest that both species have similar mediolateral stability and support surface area when visual input is removed, likely due to comparable compensatory mechanisms involving a heavier reliance on proprioceptive and vestibular inputs. However, the differences in craniocaudal (anterorposterior) stability, COP movement length, and COP speed highlight distinct postural control strategies and anatomical adaptations between bipeds and quadrupeds. Humans, with a higher COM rely more on visual input, exhibited by greater anterorposterior sway and instability when blindfolded. In contrast, dogs, with a lower COM and larger BOS, show less reliance on visual input and employ different compensatory mechanisms to maintain balance. These results underscore the importance of considering anatomical differences, sensory input reliance, and evolutionary adaptations when comparing static posturography between humans and dogs.

Future studies could benefit from including additional anthropometric and morphometric parameters, such as leg length and precise COM height, to better account for interspecies differences in postural control. In humans, the COM is relatively high in the trunk, whereas in dogs it is lower, positioned in the thoracic region. Incorporating such measurements could help clarify how anatomical differences influence sway patterns and RI, improving the interpretation of static posturography across species.

## Materials and methods

### Approval and consent

This study was conducted in accordance with ARRIVE guidelines and ethical standards for both animal and human trials. The human trial was performed in accordance with the Declaration of Helsinki and was discussed and approved by the institutional ethics committee of the Medical University of Vienna, adhering to good scientific practice guidelines and national legislation (EK No. 2261/2021). Similarly, the animal trial was approved by the Ethics and Animal Welfare Committee of the University of Veterinary Medicine, Vienna in accordance with the University’s guidelines for Good Scientific Practice guidelines and national legislation (ETK-148/10/2021). Written informed consent was obtained from all human participants as well as dog owners prior to the study.

### Sample size and inclusion criteria

In this prospective cohort study, two groups were investigated: 22 healthy young adult humans (11 women and 11 men) and 22 healthy client-owned young adult dogs (12 males—9 intact and 3 neutered; 10 females—5 spayed and 5 intact). The sample size for the human group was determined based on previously conducted studies^16,46,55–57^. To ensure homogeneity, an equal number of dogs were included in the study.

The breeds of the dogs consisted of Border Collie (*n* = 5), Labrador Retriever (*n* = 5), mixed breed (*n* = 3), Standard Poodle (*n* = 1), Flat-Coated Retriever (*n* = 1), Malinois (*n* = 1), Irish Setter (*n* = 1), Magyar Vizsla (*n* = 1), Pointer (*n* = 1), Greyster (*n* = 1), Australian Shepherd (*n* = 1), and Golden Retriever (*n* = 1).

The age of our human participants ranged (Max-Min) from 34 to 24 (30.3 ± 2.9) years, while the age of the participating dogs ranged from 6.3 to 1.4 (3.5 ± 1.5) years. In alignment with widely accepted legal and biomedical standards, we defined young adults as adults up to 39 years of age^58^. For dogs, young adults were defined based on previously published literature as individuals within the first 50% of their expected lifespan^59,60^. The body mass of the humans ranged from 105 to 50 (71.8 ± 17.7) kg, whereas the body mass of the dogs ranged from 36.0 to 13.5 (22.7 ± 6.0) kg. The body height in human groups was ranged from 193 to 152 (175.1 ± 11.0) cm and in dog group from 67.0 to 43.0 (55.4 ± 6.5) cm.

Human participants confirmed to be free from any musculoskeletal, neurological, and visual disorders. Inclusion criteria for dogs consisted of the absence of any clinical signs for musculoskeletal, neurological, or visual disorders and a minimum body weight of 10 kg to ensure accurate detection of the COP parameters by the pressure measurement plate. Each dog underwent a comprehensive clinical examination conducted by qualified veterinarians (MA, NA, CL). As a standard procedure^9,32^, this included visual gait assessment, joint palpation, and objective gait analysis including calculation of the symmetry index (SI).

### Observers

In this study, all measurements, including those of human participants and dogs, as well as the analysis of acquired data, were conducted by three observers (MA, NA, CL). All observers had a similar level of expertise in kinetic studies and data analysis and received training prior to the main study.

### Equipment

The measurement of all COP parameters in this study was done by a Zebris pressure measurement platform (FDM Type 2, Zebris Medical GmbH, Allgäu, Germany) at a frequency of 100 Hz on a flat ground. The platform was equipped with 15,360 sensors covering an area of 203 × 54.2 cm. The size of each sensor was reported to be 0.72 × 0.72 cm. To standardize the coefficient of friction, the pressure plate was covered with a 1-mm-thick non-slip rubber mat made of polyvinyl chloride. All measurement procedures were recorded with a Panasonic NV-MX500 camera (Panasonic, Kadoma, Osaka, Japan), using a standardized setup for camera positioning and angle (Fig. 3)^32^.

### Objective gait analysis of dogs

Objective gait analysis in dogs is performed to provide a precise and quantitative evaluation of their locomotor function. It helps confirm that dogs have normal gait patterns and symmetry, ensuring that any differences observed in PS are due to sensory or postural control factors rather than pre-existing gait problems. This is especially important in veterinary medicine, where subtle lameness can be difficult to detect, particularly in small dogs or those with thick coats. Objective gait analysis was based on the evaluation of the vertical GRF using a pressure measurement platform and the calculation of the SI. Before starting the procedures, the dogs were given time to move freely around the room to familiarize themselves with the measurement setup. Following this, they were guided across the pressure plate until at least five valid passes per paw were recorded. A pass was considered valid if the dog walked across the plate in a straight line without altering speed, turning its head, or pulling on the leash. The mean speed (m/s) and acceleration (m/s²) were calculated for the left forelimb. The variation in the dogs’ crossing speed had to remain within ± 0.3 m/s, with acceleration fluctuations limited to ± 0.5 m/s²^19,22,32^. The symmetrical gait was confirmed when the SI for peak vertical force (PFz) and vertical impulse (IFz) remained below 3%^19,22,32,61^. The symmetry index (SI) was calculated for both parameters (PFz and IFz) using a formula adapted from Budsberg et al. 1993^61^ and expressed as a percentage (SI%).

1 $$SIXFz\left(\mathrm{\%}\right)=abs\left(\frac{\left[XFzLLx-XFzRLx\right]}{\left[XFzLLx+XFzRLx\right]}\right)\times100$$

The XFz represents the mean value of the peak vertical force (PFz) or vertical impulse (IFz) of valid steps, LLx denotes the left front- or hindlimb, and RLx refers to the right front- or hindlimb. Perfect symmetry between the right and left front- or hindlimbs is assigned a value of 0%.

### Static posturography of humans

The measurements were conducted while participants stood upright in a bipedal stance on a flat surface under both EO and EC conditions. Participants were instructed to remain still on a pressure measurement platform, keeping their arms relaxed at their sides and their feet al.igned with their shoulders. During the EO condition, they focused on a designated spot on the wall, whereas, in the EC condition, they were asked to simulate looking at the same spot with their eyes closed. All participants were barefoot throughout the procedure. The measurements began with the EO condition, followed by the EC condition once 2 valid trials were recorded. Each trial lasted a maximum of 30s. For each condition, two valid trials were selected for analysis. A trial was considered valid if the participant maintained the instructed posture—standing silently with feet shoulder-width apart, arms at their sides, and without any head, body or hand movements—while maintaining the required focus. Each trial was assessed immediately upon completion. The recorded data were processed using a customized software, Pressure Analyzer (Michael Schwanda, version 4.8.5.0), and were low-pass filtered using a fourth-order Butterworth Filter with a cutoff frequency of 10 Hz^62^. Based on a previously published validation study, two 5 s measurements per trial were selected for statistical analysis^32^. The 5-second time windows within the measurement periods were selected based on the segments where the subject exhibited the most stable standing position without visible movement. Although healthy adults appear to maintain a steady bipedal stance, subtle postural sway is always present due to normal physiological fluctuations in balance. Observers reviewed the video recordings to identify the periods with the least visible movement and minimal postural adjustments. This approach ensures that the analyzed segment reflects the subject’s most stable posture during each trial, while allowing reproducibility by clearly defining the visual criteria used for selection. This selection was guided by the observer’s experience and captured videos. By choosing this 5-second time windows, we ensured that the analyzed window represented the most consistent and reliable posture for each subject, enhancing the accuracy of the PS assessment. The results from each trial were exported to Microsoft Excel (2016), and the final analysis was based on the average of two valid measurements per condition^16^.

### Static posturography of dogs

Static posturography in dogs was conducted following a brief rest period after objective gait analysis. The assessments took place on a level surface, where the dogs were required to stand motionless on a pressure platform with all limbs positioned perpendicular to it. Similar to procedures used in human measurements, the evaluation began with the EO condition, followed by the EC condition once valid trials were recorded. The blindfolding of the dogs for the EC condition was achieved using taped laser goggles (Laser Safety Doggles^®^, LASERVISION GmbH & Co. KG, 90766 Fürth, Germany). Each trial lasted a maximum of 20 s, and three valid trials were collected per condition. The dogs were given breaks between trials and received treats as a reward after each session. A trial was deemed valid if no movement of the body, head, tail, or paws was detected on the video recordings. The recorded data was low-pass filtered using a fourth-order Butterworth Filter with a cutoff frequency of 10 Hz and analyzed using custom software, Pressure Analyzer (Michael Schwanda, version 4.8.5.0). For each valid trial, two 5 s segments were selected for further analysis^32^. As described for humans, the same approach was applied to dogs, selecting 5-second time windows within the 20-second measurement period. The results were then exported to Microsoft Excel (2016), and the final analysis was conducted based on the average of two valid measurements per condition.

### Determination of base of support

- Base of support (BOS): BOS consisted of the area enclosed by the coordinates of the center of the feet/paws in square centimeters (cm^2^).
- Base of support length (BOS L): In this study, the BOS L was determined for human participants by dividing each foot into anterior and posterior sections. The COP was calculated for each part, and the BOS L was measured as the distance between the midpoint of the COPs of the left and right anterior foot sections to the midpoint of the COPs of the left and right posterior foot sections in cm. For dogs, the BOS L was defined as the distance between the midpoint of the COPs of the left and right forelimbs and the midpoint of the COPs of the left and right hindlimbs in cm, as illustrated in Fig. 2.
- Base of support width (BOS W): The distance was measured between the midpoint of the COPs of the left forelimb and hindlimb in dogs (or the foot sections in humans) and the midpoint of the COPs of the right forelimb and hindlimb in dogs (or the foot sections in humans), as shown in Fig. 2.

### Calculation of body COP

Pressure sensors across the plate measure load distribution from the human’s or dog’s legs/limbs. The COP is determined using weighted averages of pressure measurements based on sensor locations^32^. The following formulas apply:

2 $$COPx=\frac{{\sum}_{i}({P}_{i}\cdot{x}_{i})}{\sum{P}_{i}}$$

3 $$COPy=\frac{{\sum}_{i}({P}_{i}\cdot{y}_{i})}{\sum{P}_{i}}$$

### COP parameters

- Mediolateral displacement: Mean deviation on the lateral axis in millimeters. It was normalized to the BOS W (in millimeters) and expressed as a percentage (MLD%).
- Craniocaudal (in dogs) or anterorposterior (in humans) displacement: Mean deviation on the craniocaudal/anterorposterior axis in millimeters. It was normalized to the BOS L (in millimeters) and expressed as a percentage (CCD%).
- COP length: refers to the length of the statokinesiogram which is the length of the line that joins the points of the COP trajectory (in centimeters). It was normalized to the surface of BOS (cm^2^) and expressed as a percentage (L%).
- Average speed (AS): defined as the mean speed of the COP sway (mm/s).
- Support Surface: The area determined by an ellipse that contains 90%^63,64^ of the points of the COP trajectory. It was normalized to the BOS and expressed as a percentage (SS%).

### Romberg index (RI)

RI quantifies the influence of visual input on PS^27,46,47^. It is calculated as the ratio of the EC score to the EO score, multiplied by 100 (EC/EO × 100). A higher RI indicates a greater reliance on visual information for maintaining balance, suggesting increased postural instability. The RI was calculated for each COP parameter as follows:

4 $$\mathrm{R}\mathrm{I}=\frac{\mathrm{s}\mathrm{t}\mathrm{a}\mathrm{n}\mathrm{d}\mathrm{i}\mathrm{n}\mathrm{g}\mathrm{m}\mathrm{e}\mathrm{a}\mathrm{s}\mathrm{u}\mathrm{r}\mathrm{e}\mathrm{m}\mathrm{e}\mathrm{n}\mathrm{t}\mathrm{w}\mathrm{i}\mathrm{t}\mathrm{h}\mathrm{E}\mathrm{C}}{\mathrm{s}\mathrm{t}\mathrm{a}\mathrm{n}\mathrm{d}\mathrm{i}\mathrm{n}\mathrm{g}\mathrm{m}\mathrm{e}\mathrm{a}\mathrm{s}\mathrm{u}\mathrm{r}\mathrm{e}\mathrm{m}\mathrm{e}\mathrm{n}\mathrm{t}\mathrm{w}\mathrm{i}\mathrm{t}\mathrm{h}\mathrm{E}\mathrm{O}}\times100$$

In this study, the normalized COP values were calculated using BOS data (BOS, BOS L, and BOS W). Based on these normalized COP values, the RI for each COP parameter of each human individual and dog was determined using the following formulas:

5 $$\mathrm{R}\mathrm{I}\mathrm{M}\mathrm{L}\mathrm{D}\mathrm{\%}=\frac{\mathrm{M}\mathrm{L}\mathrm{D}\mathrm{\%}\mathrm{w}\mathrm{i}\mathrm{t}\mathrm{h}\mathrm{E}\mathrm{C}}{\mathrm{M}\mathrm{L}\mathrm{D}\mathrm{\%}\mathrm{w}\mathrm{i}\mathrm{t}\mathrm{h}\mathrm{E}\mathrm{O}}\times100$$

6 $$\mathrm{R}\mathrm{I}\mathrm{C}\mathrm{C}\mathrm{D}\mathrm{\%}=\frac{\mathrm{C}\mathrm{C}\mathrm{D}\mathrm{\%}\mathrm{w}\mathrm{i}\mathrm{t}\mathrm{h}\mathrm{E}\mathrm{C}}{\mathrm{C}\mathrm{C}\mathrm{D}\mathrm{\%}\mathrm{w}\mathrm{i}\mathrm{t}\mathrm{h}\mathrm{E}\mathrm{O}}\times100$$

7 $$\mathrm{R}\mathrm{I}\mathrm{L}\mathrm{\%}=\frac{\mathrm{L}\mathrm{\%}\mathrm{w}\mathrm{i}\mathrm{t}\mathrm{h}\mathrm{E}\mathrm{C}}{\mathrm{L}\mathrm{\%}\mathrm{w}\mathrm{i}\mathrm{t}\mathrm{h}\mathrm{E}\mathrm{O}}\times100$$

8 $$\mathrm{R}\mathrm{I}\mathrm{A}\mathrm{S}=\frac{\mathrm{A}\mathrm{S}\mathrm{w}\mathrm{i}\mathrm{t}\mathrm{h}\mathrm{E}\mathrm{C}}{\mathrm{A}\mathrm{S}\mathrm{w}\mathrm{i}\mathrm{t}\mathrm{h}\mathrm{E}\mathrm{O}}\times100$$

9 $$\mathrm{I}\mathrm{S}\mathrm{S}\mathrm{\%}=\frac{\mathrm{S}\mathrm{S}\mathrm{\%}\mathrm{w}\mathrm{i}\mathrm{t}\mathrm{h}\mathrm{E}\mathrm{C}}{\mathrm{S}\mathrm{S}\mathrm{\%}\mathrm{w}\mathrm{i}\mathrm{t}\mathrm{h}\mathrm{E}\mathrm{O}}\times100$$

In the next step, all 5 RI values of dogs and humans were statistically analyzed.

### Statistical analysis

Statistical analysis was performed using IBM SPSS version 29 (IBM, Chicago, USA). Descriptive statistics were calculated for the RI values of each group (human and dog), including means and standard deviations (SD). To compare all recorded RI COP parameters between the two groups, an Independent Samples t-test was conducted. This test was used to assess whether significant differences existed between the two groups for each RI parameter. Prior to analysis, the normal distribution of the data was verified using the Shapiro-Wilk test. Data were analyzed using a one-way analysis of variance (ANOVA) to compare the means of the measured parameters between the two groups. To evaluate the effect size of the factor, omega-squared (ω²) was calculated following the results of the ANOVA. ω² represents the proportion of variance in the dependent variable that is explained by the independent variable, corrected for sample bias. The values of ω² range from 0, indicating no explained variance, to 1, indicating that the factor explains all variance in the outcome variable. According to Cohen (1988), ω² values of 0.01 to 0.06 are conventionally interpreted as a small effect, values from 0.06 to 0.14 as a medium effect, and values of 0.14 or higher as a large effect. Statistical significance was considered at an alpha level of *p* < 0.05.

## Supplementary Information

Below is the link to the electronic supplementary material.

Supplementary Material 1

## Data Availability

The raw data supporting the findings of this article will be made available by the authors, on reasonable request by contacting the corresponding author.

## Abbreviations

AS: average speed of COP
BOS L: length of the base of support
BOS W: width of the base of support
BOS: base of support
CCD: craniocaudal (anteroposterior) displacement
COG: center of gravity
COM: center of mass
COP: center of pressure
EC: eyes closed
EO: eyes open
GRF: ground reaction force
IFz: vertical impulse
L: length of COP
MLD: mediolateral displacement
PFz: peak vertical force
PS: postural stability
RI AS: Romberg Index calculated from the average speed of COP movement
RI CCD%: Romberg Index calculated from the normalized anterorposterior (craniocaudal) displacement of COP
RI L%: Romberg Index calculated from the normalized length of COP
RI MLD%: Romberg Index calculated from normalized mediolateral displacement of COP
RI SS%: Romberg Index calculated from the normalized support surface of COP
RI: Romberg indices
SD: standard deviation
SI: symmetry index
SS: support surface of COP
