# Supplementary material for: Different strategies of bipeds and quadrupeds to maintain postural stability- a comparison of healthy humans and dogs via static posturography
Source: Sci Rep. 2026 Mar 9;16:14408. doi: 10.1038/s41598-026-42726-2 (PMC13149523; doi:10.1038/s41598-026-42726-2)
Supplement: Supplementary file 1 — Supplementary Material 1 [file 41598_2026_42726_MOESM1_ESM.pdf]

Table S1. Descriptive statistics of the investigated center of pressure values in the study for each group under eyes open (EO) and eyes closed (EC) conditions.

	**Mediolateral Displacment of Body COP (mm)**				**Craniocaudal/Anteroposterior Displacement of Body COP (mm)**				**Length of Body COP (m)**				**Average Speed of Body COP (mm/s)**				**Support Surface of Body COP (mm^2)**			
	Dog EO	Dog EC	Human EO	Human EO	Dog EO	Dog EC	Human EO	Human EO	Dog EO	Dog EC	Human EO	Human EO	Dog EO	Dog EC	Human EO	Human EO	Dog EO	Dog EC	Human EO	Human EO
1	2.24	1.96	3.35	3.15	6.35	4.43	7.56	7.04	0.08	0.07	0.03	0.04	16.34	13.55	6.36	7.47	7.03	4.13	23.86	19.62
2	1.56	2.39	3.75	3.32	3.24	4.75	7.10	4.43	0.06	0.06	0.03	0.03	11.53	12.78	6.48	6.22	3.09	7.32	22.33	9.66
3	1.67	2.33	4.59	3.09	4.81	4.48	8.59	9.84	0.09	0.08	0.05	0.05	17.51	15.48	9.22	9.50	4.71	5.48	29.94	20.90
4	3.05	3.56	5.08	5.66	8.72	5.10	5.57	8.35	0.16	0.10	0.04	0.05	32.62	20.03	7.06	10.55	17.08	11.04	20.79	38.83
5	1.79	2.55	7.46	6.91	4.72	5.11	4.25	9.08	0.10	0.08	0.04	0.05	19.05	15.42	8.19	10.85	5.24	7.17	27.79	57.09
6	2.29	2.03	5.78	4.33	6.87	4.59	8.25	4.86	0.09	0.09	0.03	0.03	17.10	18.27	6.74	5.81	5.31	4.28	44.65	20.59
7	2.05	3.02	3.97	5.11	5.84	5.77	6.76	6.94	0.09	0.08	0.03	0.03	17.15	16.26	5.92	6.84	6.13	10.17	18.79	25.53
8	1.54	2.43	6.47	5.94	3.39	4.51	6.96	6.19	0.06	0.06	0.03	0.04	11.55	11.99	6.66	7.07	4.02	6.15	31.74	38.33
9	2.76	2.78	2.95	3.49	8.12	4.78	5.88	4.51	0.16	0.12	0.03	0.03	31.49	23.22	5.32	6.96	12.80	11.40	12.14	13.03
10	2.84	2.57	7.03	7.12	6.17	5.12	10.27	8.69	0.10	0.08	0.05	0.04	19.81	16.01	9.12	8.58	12.14	7.21	58.58	54.55
11	2.42	1.97	7.28	5.26	5.94	4.22	7.94	6.12	0.08	0.07	0.05	0.05	16.56	13.71	9.31	10.45	8.01	4.81	48.86	23.05
12	3.52	2.29	8.10	3.67	6.80	5.16	6.92	7.05	0.11	0.08	0.04	0.04	22.19	15.69	8.38	7.96	8.64	5.20	47.17	21.24
13	2.98	2.66	3.26	6.73	7.65	5.36	7.67	10.85	0.08	0.08	0.03	0.04	15.70	15.11	5.33	8.96	13.48	7.36	18.70	57.05
14	2.27	2.35	5.60	5.19	4.87	5.92	6.09	8.28	0.12	0.13	0.04	0.05	23.17	25.09	7.63	10.52	6.69	7.94	26.12	32.78
15	3.39	2.83	5.91	4.18	7.67	5.28	5.98	6.57	0.10	0.11	0.03	0.03	19.58	21.84	5.41	6.21	16.64	10.37	33.50	24.99
16	2.77	1.99	7.09	4.02	6.62	4.58	8.85	7.08	0.19	0.08	0.04	0.03	38.02	16.44	7.36	6.59	8.96	5.38	58.93	19.34
17	3.42	2.43	6.53	7.16	7.25	5.56	4.75	7.07	0.17	0.07	0.04	0.04	33.59	13.85	7.85	8.82	13.10	9.71	24.64	45.21
18	2.44	3.56	6.10	6.99	7.78	6.38	7.44	6.35	0.16	0.10	0.04	0.04	31.47	19.35	7.43	7.19	10.28	12.67	43.22	49.20
19	3.01	2.20	3.05	3.16	8.23	5.89	8.48	9.51	0.08	0.12	0.04	0.05	16.15	23.79	8.30	9.79	8.74	8.30	20.78	22.52
20	2.29	3.27	2.70	3.17	5.17	4.70	5.01	6.64	0.07	0.07	0.03	0.04	13.87	14.51	6.32	8.16	6.76	9.33	11.65	14.68
21	3.25	3.42	1.93	1.60	6.36	5.46	4.05	4.08	0.11	0.09	0.02	0.02	21.01	18.69	3.91	4.39	11.77	13.04	5.50	3.31
22	1.92	2.89	3.26	3.37	5.73	6.52	3.53	8.49	0.12	0.14	0.03	0.03	24.52	27.03	5.02	5.65	7.35	11.03	9.10	18.40
Mean	2.52	2.61	5.06	4.66	6.29	5.17	6.72	7.18	0.11	0.09	0.03	0.04	21.36	17.64	6.97	7.93	9.00	8.16	29.04	28.63
SD	0.62	0.50	1.84	1.63	1.50	0.64	1.73	1.82	0.04	0.02	0.01	0.01	7.53	4.24	1.47	1.83	3.94	2.74	15.35	15.71

Table S2. Descriptive statistics of the investigated base of support values for each group under eyes open (EO) and eyes closed (EC) conditions.

	**Length of the base of the support (cm)**				**Width of the base of the support (cm)**				**Surface area of the base of the support (cm^2)**			
	Dog EO	Dog EC	Human EO	Human EO	Dog EO	Dog EC	Human EO	Human EO	Dog EO	Dog EC	Human EO	Human EO
1	47.32	50.01	11.54	11.54	18.38	17.56	20.35	22.87	863.15	876.50	234.48	263.79
2	51.78	50.82	13.34	13.13	22.15	19.41	24.48	27.32	1147.20	981.89	327.18	358.51
3	55.12	54.37	13.02	13.13	22.71	24.03	33.69	33.28	1214.75	1304.27	437.31	436.95
4	69.63	56.50	13.44	13.76	21.85	20.97	24.59	27.68	1512.66	1174.23	330.27	379.98
5	50.92	52.84	12.49	12.91	22.13	20.97	17.64	18.22	1124.21	1116.81	219.37	235.15
6	53.92	56.05	12.17	12.28	21.63	19.75	20.13	19.90	1158.63	1074.85	245.06	244.21
7	56.46	54.74	11.33	11.64	18.14	19.80	21.00	15.90	995.20	1064.72	237.35	184.90
8	50.74	50.52	12.60	12.81	15.50	18.89	13.36	17.78	783.40	961.00	167.24	227.89
9	57.11	63.67	10.80	10.38	22.97	20.31	21.84	23.97	1306.37	1305.21	235.60	248.60
10	54.26	55.72	13.55	13.34	20.81	20.84	20.54	27.74	1097.85	1153.61	277.51	369.18
11	46.62	48.82	12.70	12.60	18.87	18.85	22.25	23.93	877.13	934.10	282.40	301.49
12	51.53	47.03	13.66	13.34	22.47	17.91	17.40	19.50	1124.57	838.27	236.94	259.80
13	56.69	56.75	13.97	14.19	23.54	24.13	26.47	25.41	1318.93	1335.64	369.80	360.39
14	48.07	46.80	13.35	13.46	14.16	14.39	23.11	22.76	669.28	649.83	306.87	305.75
15	52.05	58.09	12.17	11.12	18.31	17.98	13.97	15.56	944.10	1038.64	170.11	173.11
16	45.16	40.93	12.70	12.91	16.20	18.30	18.69	20.78	730.06	667.85	236.68	267.96
17	61.07	60.03	14.50	14.50	18.07	15.78	19.28	17.62	1101.08	934.82	279.48	254.83
18	56.90	57.47	12.07	12.17	19.39	19.10	19.07	15.69	1079.16	1053.30	229.95	190.68
19	49.55	50.68	13.02	12.81	19.56	19.49	28.39	28.80	970.37	983.50	369.40	368.77
20	43.55	42.42	11.75	11.86	22.25	17.80	22.91	24.79	963.64	753.95	269.08	293.56
21	61.01	61.31	10.69	11.01	23.09	17.73	18.64	20.12	1383.20	1065.13	199.29	221.43
22	63.37	63.99	13.66	13.76	21.58	22.78	24.82	25.89	1369.62	1451.30	338.20	355.19
Mean	53.77	53.61	12.66	12.67	20.17	19.40	21.48	22.52	1078.84	1032.70	272.71	286.46
SD	6.32	6.22	1.02	1.07	2.68	2.35	4.58	4.83	220.75	208.18	67.94	72.19

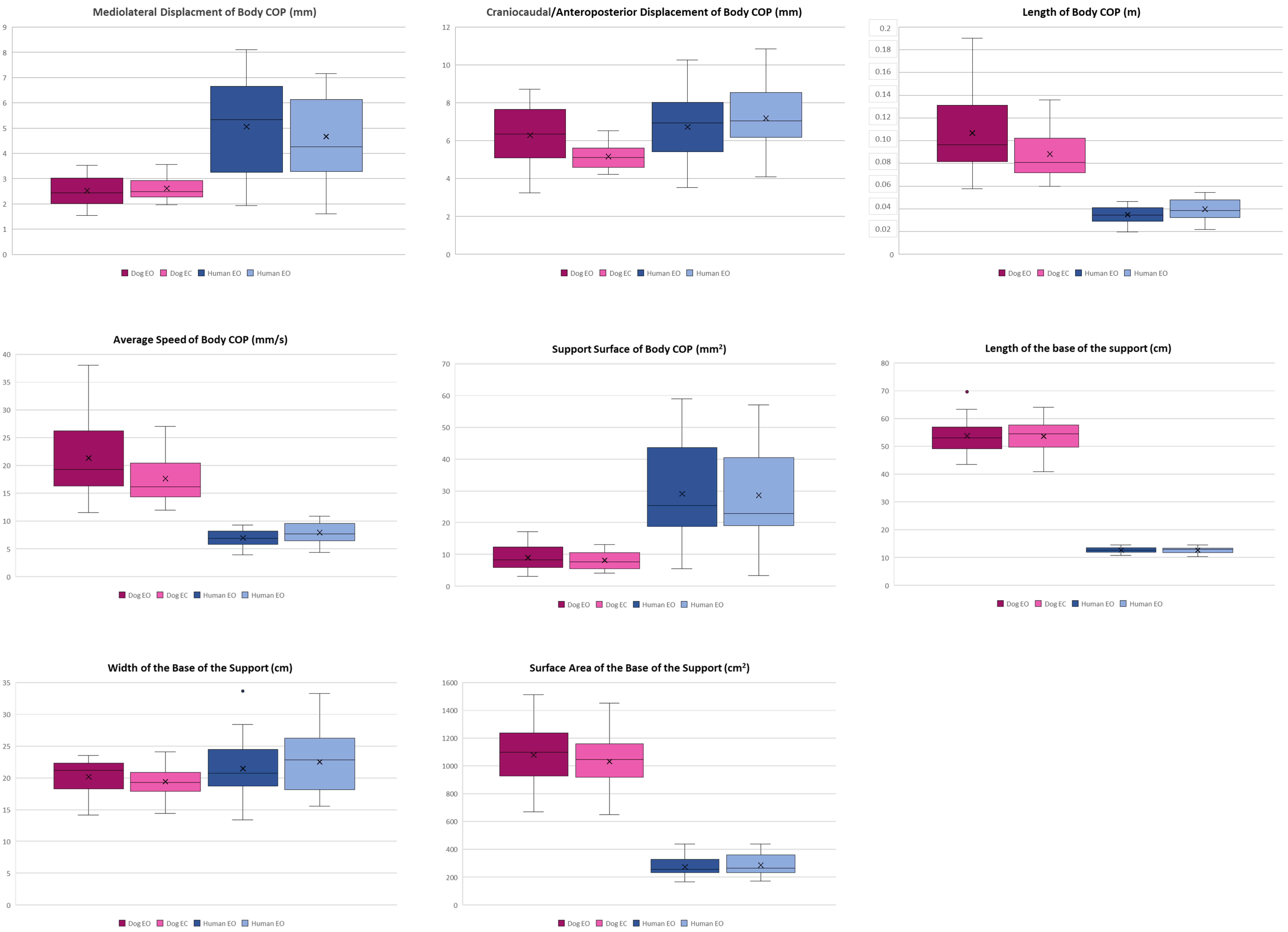

Figure S3. Boxplots of the center of pressure and base of support parameters for each group under eyes open (EO) and eyes closed (EC) conditions.
